# Safety Precautions for the Corona Mortis using Minimally Invasive Ilioinguinal Approach in Treatment of Anterior Pelvic Ring Fracture

**DOI:** 10.1111/os.12679

**Published:** 2020-04-14

**Authors:** Meng‐meng Du, Ai‐guo Wang, Xiao‐hua Shi, Bo Zhao, Ming Liu

**Affiliations:** ^1^ First Clinical Medical College Shandong University of Traditional Chinese Medicine Jinan China; ^2^ Department of Trauma Orthopaedics Zhengzhou Orthopaedic Hospital Zhengzhou China; ^3^ Department of Peripheral Blood Vessel The Affiliated Hospital of Shandong University of Traditional Chinese Medicine Jinan China

**Keywords:** Corona mortis, Minimally invasive, Pelvic fracture, Plate insertion, Safety

## Abstract

**Objective:**

To explore the safety of the corona mortis of the minimally invasive plate insertion in treatment of the anterior pelvic ring fracture by studying the relationship between the vessel and the position of plate.

**Method:**

The corona mortis was dissected out of eight adult cadavers and were simulated for the insertion of the anterior ring minimally invasive plate, and the presence of the anastomotic branch (the corona mortis) in the suprapubic branch area was observed. After the Corona mortis stripped off, the data was measured, such as the length, vessel diameter, distance from the pubic tubercle, and the maximum vertical distance between the corona mortis and the pubis. The measured data and the previous literatures were analyzed to study the morphology of the corona mortis and the position relation between the corona mortis and the placement of subperiosteal tunnel through the minimally invasive ilioinguinal approach.

**Results:**

Out of the 16 unilateral pelvises, the corona mortis were observed on 12 unilateral pelvises with an incidence rate of 75%. Amongst them, there were seven cases of vein anastomosis (incidence of 43.75%), three cases of arterial anastomosis (incidence of 18.75%), and two cases of both arterial anastomosis and vein anastomosis (incidence of 12.5%). The corona mortis length ranged between 24.5 and 37.5 mm (average of 30.7 ± 3.6 mm); the diameter ranged between 1.6 and 3.5 mm (average of 2.5 ± 0.5 mm) and the distance between the vessels and the pubic tubercle was between 53.9 and 65.2 mm (average of 59.0 ± 3.6 mm). Above the pubis, the corona mortis originated from the iliac or the inferior epigastric vessel. It crossed the pubic branch to the dorsal side of the pubis and proceeded downward to anastomize with the obturator vessels near the obturator. Toothless tweezers were used to peel and lift up the corona mortis from the pubic bone. The maximum vertical distance between the corona mortis and the pubis ranged between 8.8 and 18.3 mm (average of 12.6 ± 3.0 mm).

**Conclusion:**

The corona mortis have a high rate of incidence, with a large number of differences in the type and shape of blood vessels among patients. Following peeling, the movement between the corona mortis and pubic bone is limited. Nevertheless, the plate and bone exfoliator still passed safely. Therefore, when surgeons use the minimally invasive ilioinguinal approach to establish channels, the process of subperiosteal stripping must be performed to avoid any accidental injury.

## Introduction

The corona mortis (crown of death) is of great significance in anterior pelvis ring fracture and its treatment. The corona mortis originates from the iliac vessel or the inferior epigastric vessel to the surface of the superior branch of the pubis and anastomose with the obturator vessel near the obturator. Because of the special position of the corona mortis, extra attention should be paid to the operation of the pelvis, acetabulum fracture, and inguinal hernia through the ilioinguinal approach. In the case of an injury to the corona mortis, bleeding is difficult to control. Thus, the term “corona mortis” consists of two Latin words: “corona” used in anatomical nomenclature to designate a crown‐like eminence or encircling structure, and “mortis,” which comes from the term “mort” meaning “death.”

With the development of industry, transportation and construction in China, the occurrence of high‐energy injuries such as traffic accidents, fall injuries, and crush injuries is also increasing. In turn, pelvic fracture is more common. The anterior pelvic ring is an important part of the pelvic ring, which provides 40% of the stability of the pelvic ring. The anterior ring of the pelvis is closely related to the bladder, urethra, inguinal canal, blood vessels, and nerves. Once it is injured in a fracture or operation, it will bring unbearable pain and dysfunction, and can be life‐threatening to the patient. The injury of anterior pelvic ring is usually concentrated on the upper and lower branches of the pubis. Patients who are considered to have poor closed reduction by imaging diagnosis often need surgical incision to completely expose the fracture end for fixation.

The ilioinguinal approach to treat fractures of the pelvis was first introduced by Letoumel^1^ in 1960. Though this approach has been successful in treating such fractures, but it is also associated with a few shortcomings: the surgical trauma and the stimulation of the blood vessels and nerves during the separation of the middle window greatly influences the outcome of the surgery. Thus, in recent years, scholars at home and abroad have proposed many modified surgical approaches^2^, ^3^, ^4^, ^5^. In addition, further approaches have been proposed, such as the Stoppa approach, the anterior combined endopelvic approach, and the Pfannenstiel approach^6^, ^7^, ^8^. Grubor *et al*.^9^ suggest that external fixation should be the first choice for Tile type B and C_1_ fractures, while internal fixation or internal fixation combined with external fixation should be used for C_2_ and C_3_ fractures. After weighing up the advantages and disadvantages, doctors should adopt safe and effective minimally invasive methods for fixation according to their own capabilities and technical conditions.

The new minimally invasive ilioinguinal approach has been favored lately as it is able to achieve a better outcome without dissecting the middle window^10^. It can also reduce trauma and operation time. However, the corona mortis often crosses over the surface of the superior pubic branch under the middle window. It is observed that the corona mortis plays an important role in the surgery of anterior ring fracture, especially in the minimally invasive ilioinguinal approach.

Angiography is the most correct and practical method in diagnosis of the corona mortis before operation. On the one hand, when peeling off this area during operation, injury to the corona mortis can be avoided more accurately. On the other hand, once the corona mortis is injured, it can be accurately exposed and hemostasis according to the predicted position of the injured blood vessel. However, in China, angiography is not feasible for some hospitals and individuals with limited conditions. Therefore, the study of morphology is particularly important. Much previous related research on the morphology of the corona mortis is found; however, this literature does not examine large enough sample sizes to summarize all the situations, and there are only two related studies in China. In addition, there is no research on the space under the corona mortis. Through the previous morphological study of the corona mortis, it is found that the risk of injury to the corona mortis is indeed high, and cannot be completely avoided in both the traditional approach and the minimally invasive approach. Therefore, the space under the corona mortis should be discussed to ensure the correct and sufficient blunt separation and safety of the corona mortis.

The current study collects the presence, dimensions, position, and lower space of the corona mortis. The measured data are analyzed and compared with previous literature in order to: (i) analyze of differences and consistencies with other research results; (ii) provide a valuable reference for big data research of the corona mortis in the future; (iii) study the safe range of operation by morphology of the corona mortis; and (iv) design a procedure which ensures the safety of the corona mortis without opening the middle window.

## Materials and Methods

### *General Information*

#### *Research Object*

Eight formaldehyde, fixed, normal adult cadavers were used in the study, which included five males and three females.

Inclusion criteria: (i) all pelvises and surrounding tissues were well preserved; (ii) all cadavers were simulated for the insertion of the minimally invasive plate for the anterior ring; (iii) the length, vessel diameter, distance from the pubic tubercle, and maximum vertical distance between the corona mortis and the pubis were measured; (iv) the measured data were analyzed and compared with previous literature; and (v) this study was approved by our institutional review board.

Exclusion criteria: (i) obvious deformity of pelvic appearance; (ii) destructive lesions and injuries in bone, nerve, and blood vessels.

#### *Tools and Equipment*

Large and small scalpel handles, large and small blades, hemostatic forceps, needle holder, periosteal dissector, toothless tweezers, medical rubber gloves, bendable model of 20‐hole steel plate, Vernier caliper, Sony digital camera.

### *Method*

The skin and subcutaneous tissues of the 16 unilateral pelvises were cut layer by layer from the anterior edge of the pubis to the anterior superior iliac spine. Afterward, the inguinal ligament was cut to strip the iliopsoas muscle along the internal iliac plate, and the external iliac arteries and veins. Then the femoral nerves were carefully separated. Thereafter we incised the iliopubic fascia and pulled the spermatic cord (or circular ligament of the uterus) on the medial side, followed by cutting off the rectus abdominis muscle on the pubic bone to expose the deeper structures. We then looked for the corona mortis along the deep surface of the external iliac vessel or the inferior vessel of the abdominal wall. The passage below the periosteum of the corona mortis was established along the pubic branch before the plate was inserted.

### *Measurement*

The following parameters were measured by two observers:

#### *Presence of the Corona Mortis*

Observe the existence of the corona mortis on each unilateral pelvis, and, if it exists, observe the type of the corona mortis. This is related to the rate of the corona mortis encountered during blunt separation and plate insertion.

#### *Dimensions of the Corona Mortis*

**Figure 1:**
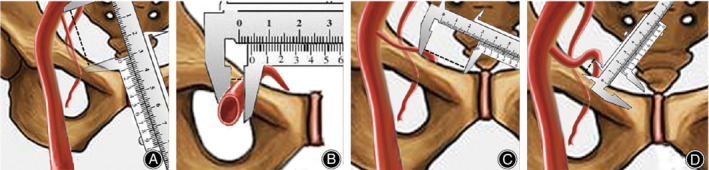
Diagram of measurement method: (A) After stripping off the corona mortis, we straightened the blood vessel and used outer measuring claw of the vernier caliper to measure the length of the corona mortis; (B) Measured the diameter of the corona mortis; (C) Measured the distance from the corona mortis to the pubic tubercle; (D) Use dinner measuring claw of the vernier caliper to measure the maximum vertical distance between corona mortis and the plate on the surface of the pubis.

The dimensions include the length and diameter of the corona mortis artery. The length of the corona mortis is related to the complexity of its cross route which can lead to vascular malformation (Fig. 1A). The larger the diameter of the blood vessel, the more likely the blood vessel is to be injured and the bleeding may be more (Fig. 1B).

#### *Position of the Corona Mortis*

The position includes the origin and extent of the corona mortis, more specifically the distance from the corona mortis to the pubic tubercle. The specific location can determine whether the medial incision using the minimally invasive approach can directly see the corona mortis, which is also helpful for preoperative planning without angiography (Fig. 1C).

#### *Space below the Corona Mortis*

The plate is inserted after removing the corona mortis and observing that the maximum vertical distance between the corona mortis and the plate exists. The triangle, which consists of the pubic branch and the corona mortis, is the lower space, the size of which is positively related to the maximum vertical distance and is required to be larger than the size of the subperiosteal tunnel (Fig. 1D).

Each numerical value was measured three times, with the average value recorded.

## Results

### *Presence of the Corona Mortis*

Out of the total 16 unilateral pelvises, corona mortis was observed on 12 unilateral pelvises with an incidence of 75%. Amongst them, seven cases showed anastomosis with an incidence of 43.75%, three cases presented with arterial anastomoses with an incidence of 18.75%, and two cases showed both arterial anastomosis and vein anastomosis with an incidence of 12.5%.

### *Dimensions of the Corona Mortis*

The length of the corona mortis ranged between 24.5 to 37.5 mm (mean, 30.7 ± 3.6 mm), and the diameter of the corona mortis ranged between 1.6 to 3.5 mm (mean, 2.5 ± 0.5 mm).

### *Position of the Corona Mortis*

**Figure 2:**
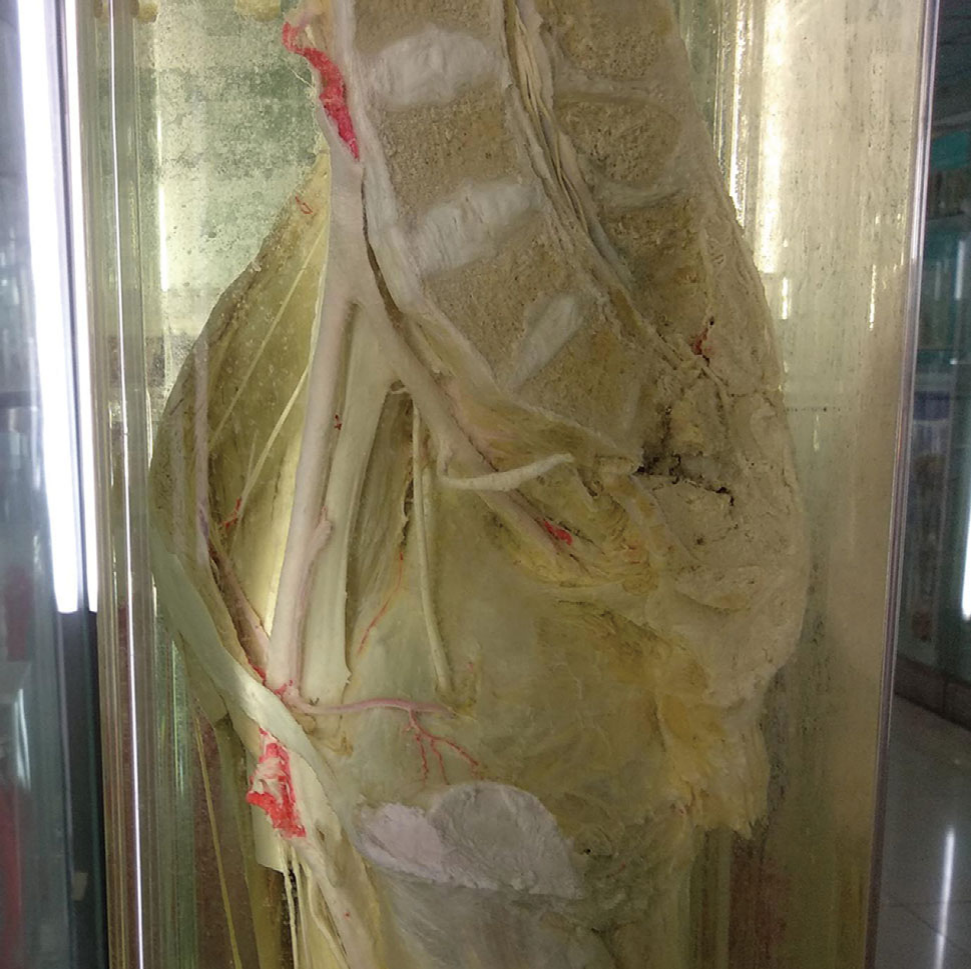
The obturator vessels and the external iliac vessels form the anastomotic branches on the dorsal side of the pubis.

**Figure 3:**
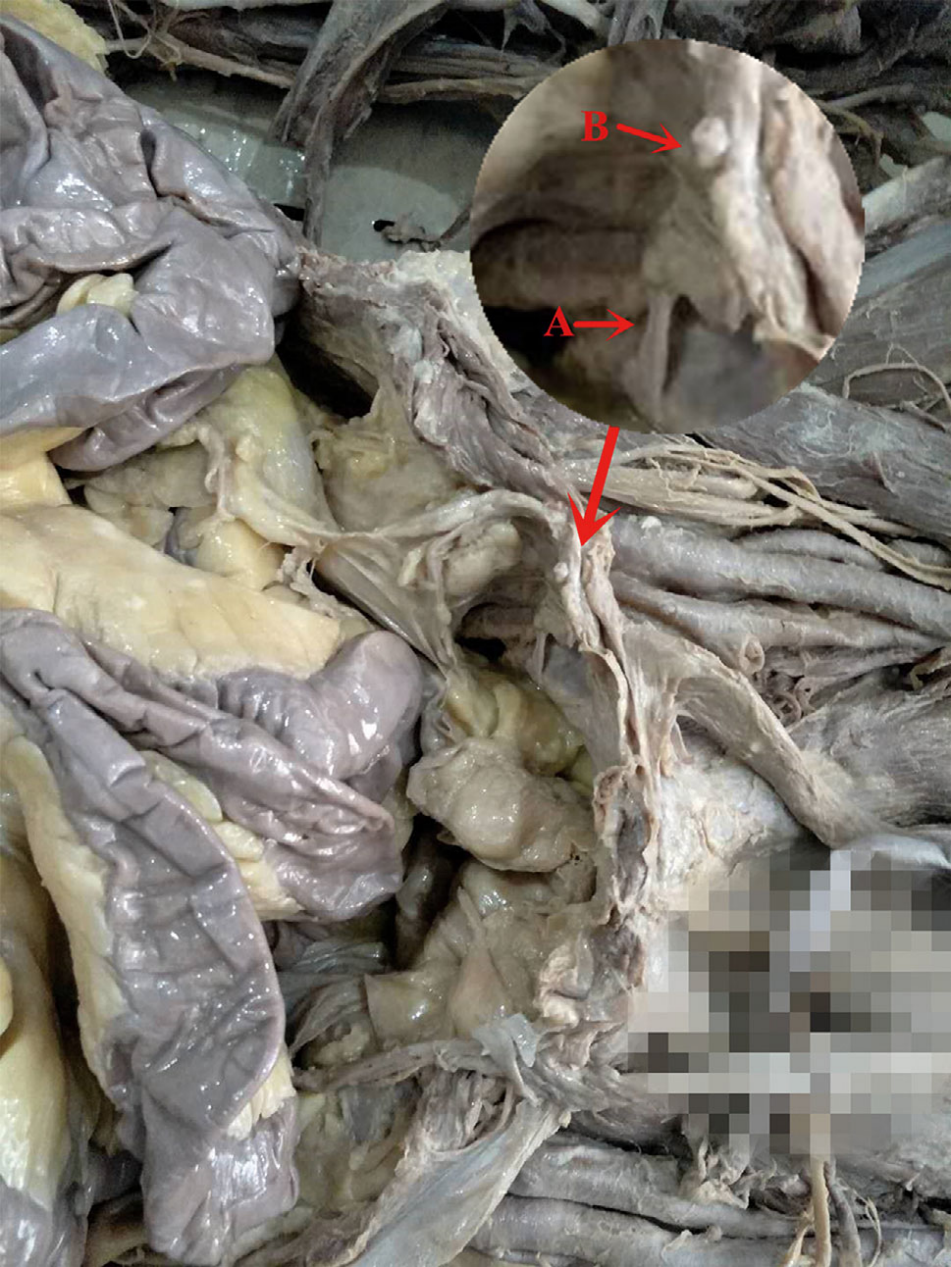
The photograph of anatomical general view: (A) The corona mortis; (B) The inferior epigastric vessel.

**Figure 4:**
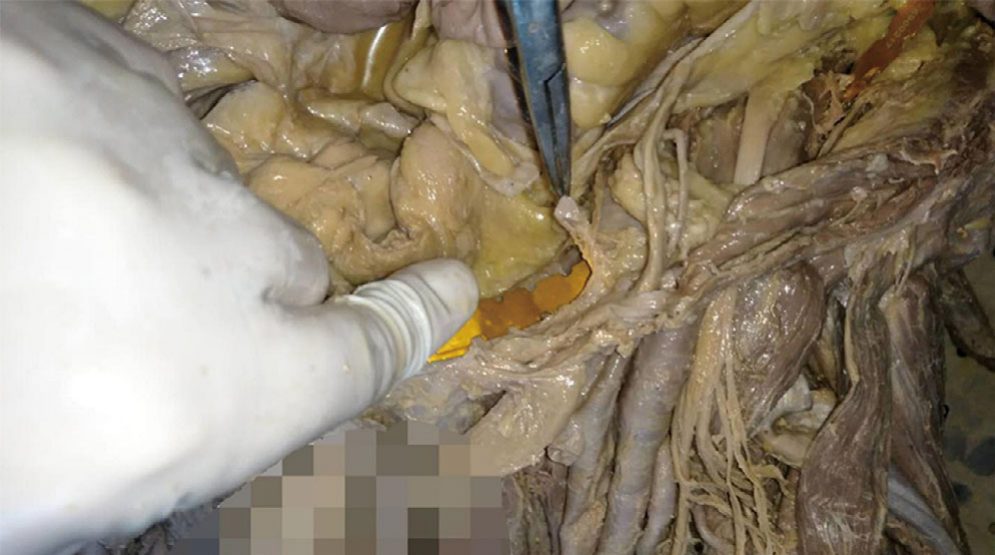
The plate model was inserted to measure the vertical distance between the anastomotic branch and the plate model.

**Figure 5:**
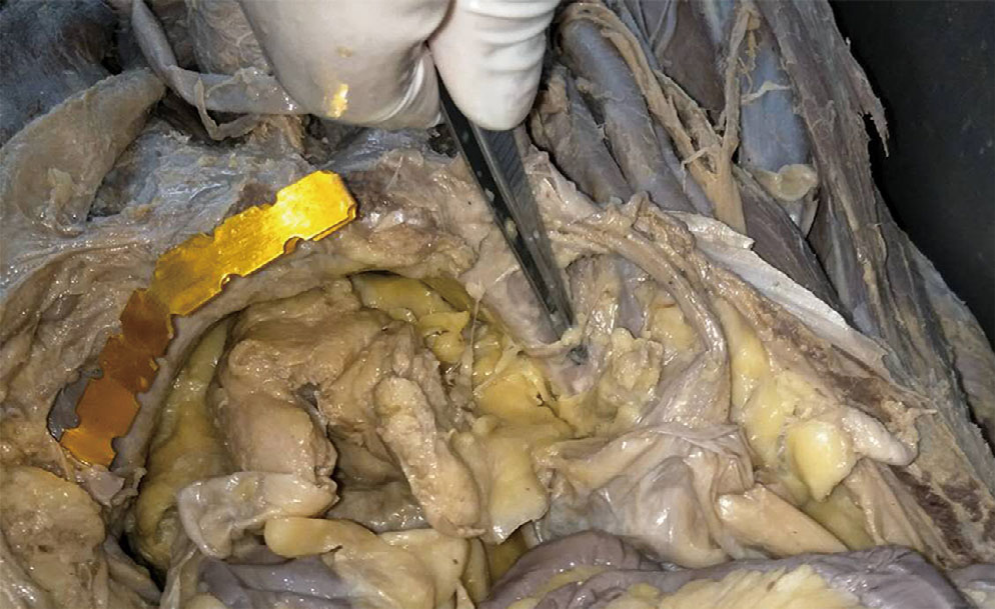
The bilateral venous anastomotic branches were present.

Above the pubis, the corona mortis originated from the iliac or the inferior epigastric vessel. It crossed the pubic branch to the dorsal side of the pubis and proceeded downward to anastomize with the obturator vessels near the obturator. The distance between the corona mortis and the pubic tubercle was 53.9 to 65.2 mm, with an average of 59.0 ± 3.6 mm (Figs 2, 3, 4, 5).

### *Space Below the Corona Mortis*

**Figure 6:**
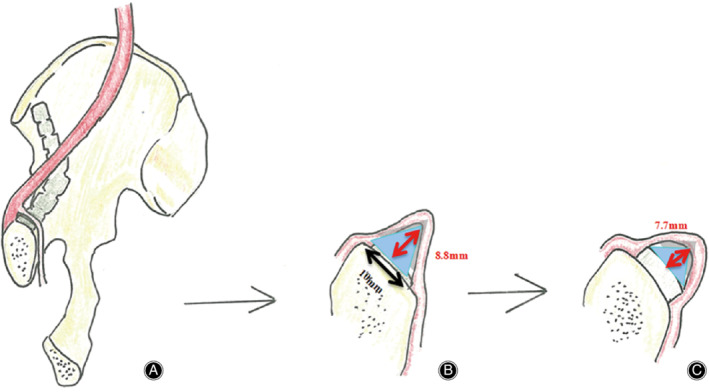
(A) The corona mortis straddles over the pubis; (B) Insertion of a 1 mm thick and 10 mm wide plate model or periosteal stripper; (C) Insertion of a 3.5 mm thick and 10 mm wide plate.

The corona mortis was removed and lifted vertically from the pubis with toothless forceps and we observed that the maximum vertical distance between corona mortis and the plate on the surface of the pubis ranged between 8.8 and 18.3 mm, with an average distance of 12.6 ± 3.0 mm (Fig. 6).

## Discussion

### *Significance of the Corona Mortis in Fracture of the Anterior Pelvis Ring*

It is known that the external iliac vessel sends a branch to the rectus abdominis muscle above the pubis to form the inferior epigastric vessel, and the later are often anastomosed with the subpubic obturator vessel to form the anastomotic branch, which is named the corona mortis^11^. Due to the absence of a sacroiliac complex, the anterior ring is weaker than the posterior ring and is more likely to be fractured when subjected to external force. If there is a corona mortis on the injured side of pelvis, it is very easy to be damaged during a fracture or an operation. Once a blood vessel is broken, it is retracted back into the pelvic cavity with a large amount of bleeding; it is difficult to find the broken end, and the resulting blood loss is difficult to control, which can be life‐threatening in some cases^12^.

Previously, the corona mortis was thought to be a variation of obturator vessels or a branch of its accessory vessels, but now studies have confirmed that no matter the source of the blood vessels, if it enters the obturator tube and becomes the main blood supply vessel, it should be treated as the corona mortis^13^, ^14^.

The importance of the corona mortis in abdominal and pelvic surgery is critical but there have been conflicting results on the incidence of the corona mortis. A study by Tornetta *et al*. on the incidence of corona mortis and its shape revealed that the arterial anastomosis was found in only 28.5% (28 of 98) of patients^15^, while Darmanis *et al*. reported its incidence in 83% of patients^16^. Moreover, other studies observed racial differences in the incidence of corona mortis^17^.

### *Relationship Between the Corona Mortis and Minimally Invasive Plate Approach*

Corona mortis injuries are mainly caused by the following two factors: first, an external force injury, such as fractures of the pelvis and acetabulum caused by violence. Garrido‐Gomez *et al*. showed that during the conservative treatment of the pubic branch fracture, there was a continuous decrease of the blood pressure of one patient decreased and the reason behind such an increase was found to be due to the corona mortis avulsion as observed by arteriography^18^. The second factor is iatrogenic injury. If the surgeon does not have full mastery of the anatomical structure involved in the operation, or the patient has an abnormal vascular system, the corona mortis can be accidentally injured during the exposure of the pubic or acetabular anterior column fracture^19^.

In the ilioinguinal approach, the vessels and the nerves around the ilioinguinal region are well protected and not easily injured during the operation because of their clear position. Another approach to preserve the corona mortis is the anterior combined endopelvic approach, which enables the direct visualization of the corona mortis as to coagulate and tie it^7^.

However, as the anatomy of the corona mortis is not well known, or if the surgeon is not familiar with the anatomy of the corona mortis, it is easy to cause vascular injury. Many researchers believe that although the corona mortis veins are more likely to be injured during surgery, they are far less important than the corona mortis arteries. However, as the veins have a fragile vessel wall and a larger diameter, it is more difficult to stop bleeding following a vein injury than an artery injury. Moreover, the location and shape of the corona mortis arteries are detected by digital subtraction angiography (DSA) before operation, to reduce the possibility of injury. Moreover, no matter which surgical method is used, as long as there are blood vessels found on the surface of the pubic ligament during an operation, it is always imperative to first suture the bleeding vessel and look for other ones which might be bleeding before proceeding further in the operation for a successful outcome.

We observed the incidence of corona mortis in 75% of the patients, similar to previous reports, which is a significant critical factor in the process of surgery undertaken^11^, ^20^, ^21^. The diameter of the corona mortis observed were between 1.6 and 3.5 mm, with an average of 2.5 ± 0.5 mm, which was of the same order of magnitude as that of the carotid artery, radial artery, and posterior tibial artery. The mean diameter of the radial artery is about 2.94 mm^22^, which can easily produce a lethal amount of bleeding, and it is more easy to control bleeding from such vessels than from venous anastomotic branch with their fragile vessel wall and longer vessel length, thus, it is more likely that the venous anastomotic branch should be more likely to be injured than the arterial anastomotic branch. Furthermore, because of the fast blood flow and high pressure of the arterial anastomotic branches, it is more difficult to stop the arterial bleeding.

Compared to the venous branches, the arterial anastomotic branch has a shorter path and greater elasticity than the pubic branch, so it is easier to retract back to the pelvis after fracture but more difficult to find and ligate blood vessels by pressing the bleeding point. So, we believe that the two kinds of blood vessels are equally importance as failure to effectively stop bleeding after rupture may lead to hemorrhagic shock, which can be life‐threatening. The mean distance between corona mortis and the pubic tubercle is 53.9 to 65.2 mm (mean, 59.0 ± 3.6 mm) and the 2–3 cm medial incision of the minimally invasive plate approach is done about 2 cm outside the pubic tubercle. Therefore, the corona mortis is often located on the lateral side of the medial incision and is unlikely to be seen in surgical fields.

Upon peeling off the blood vessels on the cadaver, the maximum vertical distance between the corona mortis and the plate model on the upper surface of the pubis was observed to be between 8.8 and 18.3 mm. The corona mortis and the pubis form an isosceles triangle and the maximum vertical distance is the height of the triangle, thus the plate model’s bottom edge has a width of about 10 mm (Fig. 6). The thickness of the plate is generally between 1 and 2 mm, and the width is about 10 mm. The periosteal stripping width of the pelvis is between 10 and 12 mm. Therefore, although the range of motion between the corona mortis and the superior branch of the pubis is not large, even after stripping to fit the required width of the plate, the periosteal exfoliator of a 12 mm width and the plate of a 10 mm width is still able to pass safely. But when we penetrate the inner and outer incision, it is necessary to peel off the periosteum from the pubis to form a tunnel under the periosteum to ensure that the corona mortis will not be injured during the insertion of the plate. However, the data from anatomy cannot be not entirely equal to the clinical data. Due to rigor mortis, the hip of the cadaver specimen is in the extension position, and the activity of surrounding tissue is poor.

In the process of clinical operation, the hip of patients is in a state of flexion, and the surrounding tissue of the anterior pelvic ring is more relaxed. Because of the suitable position and relaxed surrounding tissue, the space under the corona mortis is likely to be larger. If the corona mortis has been injured during the peeling process, the medial incision can be extended outward by 2–3 cm, then the corona mortis can be validated after exposure. Thus, as Italian scholar Beatrice Sanna said, all surgeons operating in the retropubic region should have a thorough understanding of the anatomical characteristics and surgical implications of a corona mortis^23^. Heterotopic ossification is a complication of minimally invasive surgery which is higher than that of traditional surgery. Some operative skills can be adopted in operation to ensure anatomical reduction to achieve results similar to those of traditional surgery, such as prying reduction and reduction after enlarged deformity. The heterotopic bone can be treated with therapy, such as indomethacin, radiation therapy, shock waves, and a second operation to recover joint function^24^.

When conditions permit, it is best for doctors to determine the location and the shape of the corona mortis by angiography before operation. This is so that, during the peeling process to this area, injury to the corona mortis can be more avoided and, even if the vessel is injured, can be accurately exposed to stop bleeding according to the location of the injured blood vessel.
